# Overworked Matrons: Educate the Committees

**Published:** 1908-01-11

**Authors:** 


### EDITOR'S LETTER-BOX.

### OVERWORKED MATRONS; EDUCATE THE COMMITTEES.

Sir,—Your article on "Overwork in Regard to Nursing Administration," in last week's issue, is most timely, and should engage the public attention. I know from my friends who have obtained matrons' posts that your statements are correct. In every department. greater efficiency, scientific accuracy and detail, nicety of management are expected, and rightly so, but still at the hands of one woman. However talented she may be, she cannot possibly increase her powers of work. This is felt more severely in the small hospitals. The older members of the committees cannot grasp modern requirements and they feel as things were managed comfortably enough in the past they can continue to be so. I know of a case where the matron laid before her committee an account of her daily duties, showing that from 8 a.m. until 10 p.m. she had no leisure moment, meals were often curtailed, all necessary correspondence was conducted up to midnight, and often afterwards, and she had difficulty in getting out more than once a week. Her suggestions of reform were met by the statement, "That the committee saw no reason to relieve the matron of any of her accustomed duties"! I know they would plead lack of funds, but why benefit one class at the expense of another. During the past year this same hospital has received more patients and brought its teaching and household management into a greater state of efficiency than ever before, and all at the expense of the health and happiness of the resident members of its staff.

I fear your remedy of "resignation" is not feasible on practical grounds. Perhaps a few who take hospital posts have private means, but in most cases a woman has herself to support, relatives to help, and her future to provide for. If she has been lucky she may find herself at, say, thirty-five years of age, earning from £70 to £80. Her only chance of promotion and a better salary in the future is to continue in her present position however arduous. It would certainly be impossible to obtain another post from retirement. Also, in the present condition of the nursing world, did she retire there would be numberless applicants for the post. The only hope of improvement is in an educated public opinion on the matter. I enclose my card, and remain,

Yours truly,

A Sister of a London Hospital.

